# Supplementary material for: Kinetics Growth and Recovery of Valuable Nutrients from Selangor Peat Swamp and Pristine Forest Soils Using Different Extraction Methods as Potential Microalgae Growth Enhancers
Source: Molecules. 2021 Jan 27;26(3):653. doi: 10.3390/molecules26030653 (PMC7866033; doi:10.3390/molecules26030653)
Supplement: Supplementary file 1 [file molecules-26-00653-s001.pdf]

molecules-1075625

Kinetics growth and recovery of valuable nutrients from Selangor Peat Swamp and Pristine Forest soils using different extraction methods as potential microalgae growth enhancers

Supplementary Figure S1

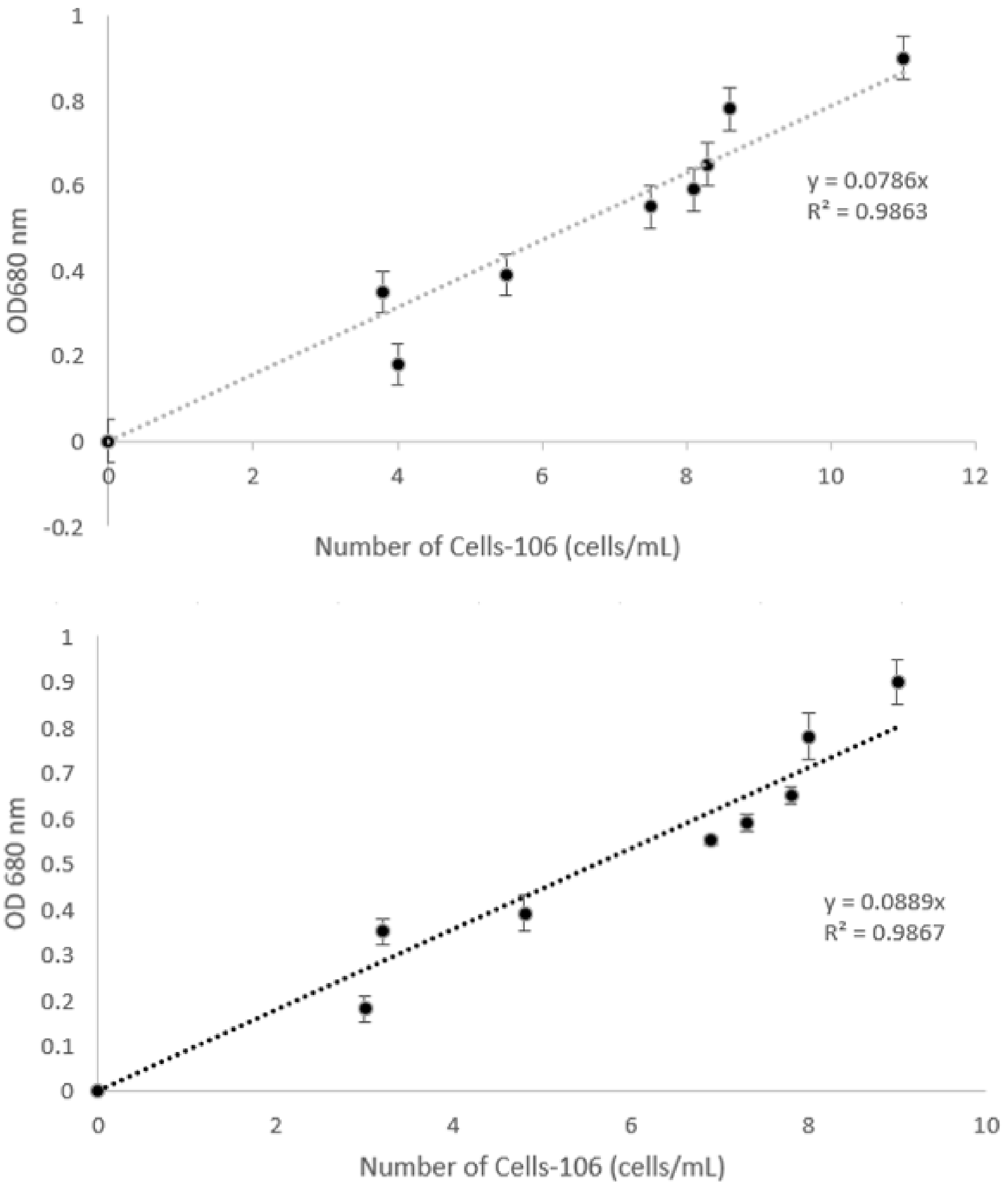

Figure S1: Correlation between optical density and number of cells. A) *C. vulgaris* and B) *N. oceanica.* Error bars represent standard deviation (n = 3)

Supplementary Figure S2

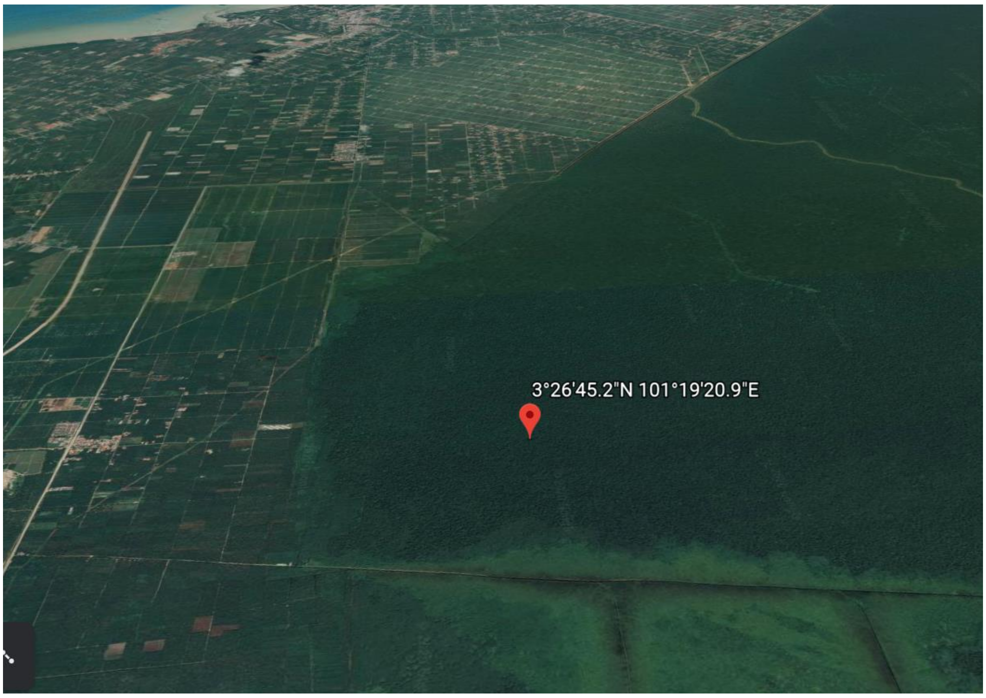

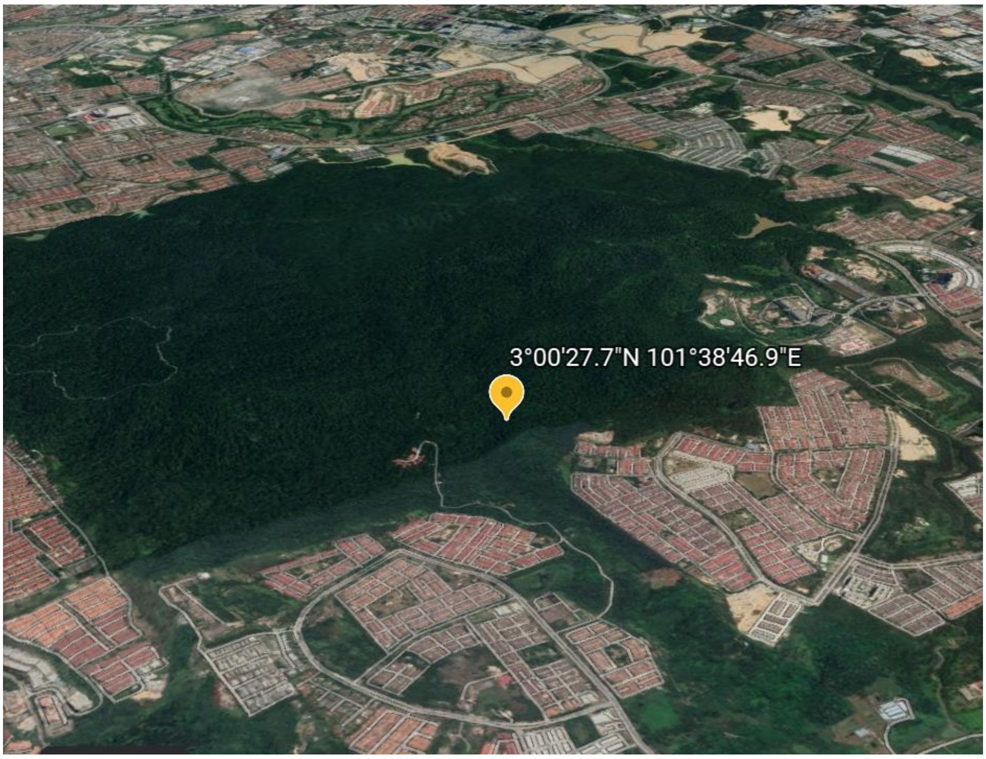

Figure S2: Map of sampling sites in this study A) Raja Musa Forest Reserve (RMFR) (3°26'45.2"N 101°19'20.9"E) and B) Ayer Hitam Forest Reserve (AHFR) (3°00′′27.7′′N 101°38′46.9′′E), Selangor Malaysia

Supplementary Table S1

Statistic Data

Data for Figure 1,2 ,3 Total dissolved nitrogen (TDN), total dissolved phosphorus (TDP) and dissolved organic carbon (DOC) concentrations in soil extracts with natural extractions.

AH (DOC)		$t(5)= 73.12$, $p < 0.0005$
RM (DOC)		$t(5)= 17.88$, $p < 0.0005$
AH (TDN)	NE1h & RT4h	$t(2)= 0.277$, $p > 0.05$
	NE1h & RT24h	$t(2)= 0.894$, $p > 0.05$
	NE4h & RT24h	$t(2)= 0.600$, $p > 0.05$
RM (TDN)	NE1h & RT4h	$t(2)= -1.292$, $p > 0.05$
	NE1h & RT24h	$t(2)= -1.305$, $p > 0.05$
	NE4h & RT24h	$t(2)= 0.197$, $p > 0.05$
AH (TDP)	NE1h & RT4	$t(2)= 0.742$, $p > 0.05$
	NE1h & RT24h	$t(2)= 0.742$, $p > 0.05$
	NE4h & RT24h	$t(2)= 0.000$, $p > 0.05$
RM (TDP)	NE1h & RT4h	$t(2)= 0.862$, $p > 0.05$
	NE1h & RT24h	$t(2)= 0.862$, $p > 0.05$
	NE4h & RT24h	$t(2)= -0.070$, $p > 0.05$

Data for Figure 1,2 ,3 Total dissolved nitrogen (TDN), total dissolved phosphorus (TDP) and dissolved organic carbon (DOC) concentrations in soil extracts autoclave extractions.

AH (DOC)	ANOVA & post hoc	$p < 0.0005$, $f = 106.234$
RM (DOC)	t-test	$t(11)= 8.378$, $p < 0.0005$
AH (TDN)	ANOVA & post hoc	$p < 0.0005$, $f = 121.600$
RM (TDN)	ANOVA & post hoc	$p < 0.0005$, $f = 39.571$
AH (TDP)	ANOVA & post hoc	$p = 0.036$, $f = 5.107$
RM (TDP)	ANOVA & post hoc	$p = 0.013$, $f = 7.956$

Data for Table 1. Ratios of concentrations of TDN, TDP and DOC in Raja Musa Forest Reserve (RMFR) to Ayer Hitam Forest Reserve (AHFR).

RM:AH (DOC)	ANOVA & post hoc	$p < 0.0005$, $f = 102.001$
RM:AH (TDN)	ANOVA & post hoc	$p < 0.05$, $f = 5.562$
RM:AH (TDP)	ANOVA & post hoc	$p < 0.05$, $f = 3.650$

Data for Table 2. Ratios of DOC to TDN and TDP, and TDN to TDP for RMFR and AHFR using different extraction methods.

C:N (AH)	ANOVA & post hoc	$p < 0.05$, $f = 7.543$
C:N (RM)	ANOVA & post hoc	$p < 0.05$, $f = 4.687$
C:P (AH)	ANOVA & post hoc	$p < 0.05$, $f = 3.573$
C:P (RM)	ANOVA & post hoc	$p > 0.05$, $f = 2.280$
N:P (AH)	ANOVA & post hoc	$p > 0.05$, $f = 0.744$
N:P (RM)	ANOVA & post hoc	$p > 0.05$, $f = 2.555$

Data for Figure 7: Optical Density at 680 nm of C.vulgaris and *N.oceanica* in control, media + 105 °C, media + 105°C twice, media + 121 °C, media + 121 °C twice and media + 24 hour at (A) RM SE and (B) AH SE. Error bars represent standard deviation ($n = 3$)

C. vulgaris (AH)	ANOVA & post hoc	$p > 0.05$, $f = 0.473$
C. vulgaris (RM)	ANOVA & post hoc	$p > 0.05$, $f = 0.559$
N. ocenica (AH)	ANOVA & post hoc	$p < 0.05$, $f = 5.972$
N. ocenica (RM)	ANOVA & post hoc	$p > 0.05$, $f = 0.794$

Data for Table 3: The maximum OD of *N. ocenica, C. vulgaris* on control, 105 °C, 105 °C twice, 121 °C, 121 °C twice and 24 hours' soil extraction (SE) from *Raja Musa* Forest Reserve (RM) and *Ayer Hitam* Forest Reserve (AH)

C. vulgaris (AH)	t-test	$t(5) = 13.137$, $p < 0.0005$
C. vulgaris (RM)	t-test	$t(5) = 7.359$, $p < 0.005$
N. ocenica (AH)	t-test	$t(5) = 4.882$, $p < 0.05$
N. ocenica (RM)	t-test	$t(5) = 8.677$, $p < 0.0005$

Data for Figure 8: Specific growth rate, µ of *N. ocenica, C. vulgaris,* in control, media + 105 °C, media + 105 °C twice, media + 121 °C, media + 121 °C twice and media + 24 hour at (A) RM SE and (B) AH SE.

C. vulgaris (AH)	ANOVA & post hoc	$p > 0.05$, f = 0.425
C. vulgaris (RM)	ANOVA & post hoc	$p > 0.05$, f = 1.393
N. ocenica (AH)	ANOVA & post hoc	$p > 0.05$, f = 1.559
N. ocenica (RM)	ANOVA & post hoc	$p > 0.05$, f = 0.650

Data for Table 4: The division rate, *k* of of *N. ocenica, C. vulgaris* on control, 105 °C, 105 °C twice, 121 °C, 121 °C twice and 24 hours' soil extraction (SE) from *Raja Musa* Forest Reserve (RM) and *Ayer Hitam* Forest Reserve (AH)

C. vulgaris (AH)	ANOVA & post hoc	$p > 0.05$, f = 0.425
C. vulgaris (RM)	ANOVA & post hoc	$p > 0.05$, f = 1.392
N. ocenica (AH)	ANOVA & post hoc	$p > 0.05$, f = 1.694
N. ocenica (RM)	ANOVA & post hoc	$p > 0.05$, f = 0.651
